# Ubiquinol Improves Symptoms in Children with Autism

**DOI:** 10.1155/2014/798957

**Published:** 2014-02-23

**Authors:** Anna Gvozdjáková, Jarmila Kucharská, Daniela Ostatníková, Katarína Babinská, Dalibor Nakládal, Fred L. Crane

**Affiliations:** ^1^Pharmacobiochemical Laboratory of 3rd Medical Department, Medical Faculty, Comenius University in Bratislava, Sasinkova 4, 811 08 Bratislava, Slovakia; ^2^Institute of Physiology, Medical Faculty, Comenius University in Bratislava, Sasinkova 4, 811 08 Bratislava, Slovakia; ^3^Department of Biological Science, Purdue University, W. Lafayette, IN 47901, USA

## Abstract

*Background*. Autism is a spectrum of neurodevelopmental disorders with manifestation within 3 years after birth. Manifestations of autism include behavior problems (hyperactivity, toys destruction, self-harm, and agression) and sleep and eating disorders. Etiology of autism is poorly understood. Oxidative stress and antioxidants can participate in pathobiochemical mechanisms of autism. *Methods*. Twenty-four children, aged 3–6 years, with autism according to the DSM IV criteria and using CARS were included in the study. Concentrations of CoQ_10−TOTAL_, **γ**- and **α**-tocopherol, **β**-carotene, and lipid peroxidation were determined in plasma before and after three months of supportive therapy with ubiquinol at a daily dose 2 × 50 mg. Data on behavior of the children were collected from parents at the same time. *Results*. Ubiquinol supportive therapy improved symptoms in children with autism, as communication with parents (in 12%), verbal communication (in 21%), playing games of children (in 42%), sleeping (in 34%), and food rejection (in 17%), with CoQ_10−TOTAL_ plasma level above 2.5 *μ*mol/L. *Conclusions*. Beneficial effect of ubiquinol in children with autism has been demonstrated for the first time. We assume that plasma concentration of CoQ_10−TOTAL_ and lipid peroxidation could be used as relevant biomarkers of ubiquinol supportive therapy.

## 1. Introduction

Autism Spectrum Disorders (ASDs) are neurodevelopmental disorders with manifestation within three years after birth,occurring in male and in female in ratio of 4 : 1. ASDs manifestations of autism include behavior problems (hyperactivity, toys destruction, self-harm, and aggression) and sleep and eating disorders, which may result from an interaction between genetic, environmental, and immunological factors [1].The evidence suggests that various factors may be involved in the etiopathogenesis of autism, such as oxidative stress, antioxidant defence systems, brain mitochondrial dysfunction, impaired immune responses, environmental toxins, and genetic disorders [2–5]. However, etiology of autism is poorly understood.

Potential mechanisms that may link oxidative stress to neuronal dysfunction, clinical symptoms, and pathogenesis of autism include membrane lipid abnormalities, decreased cellular membrane fluidity, immune and inflammatory response, increased excitotoxicity, abnormal iron and copper metabolism [6], imbalance in homocysteine/methionine metabolism, increased nitric oxide and xanthine oxidase, and mitochondrial dysfunction and abnormal energy metabolism [4, 6, 7]. Antioxidant defence systems in autism can be activated [8] or decreased [9]. Total antioxidant status was reduced in Asperger individuals (mild subtype of autism) compared with healthy controls [10]. Levels of major antioxidant serum proteins, namely, transferrin (iron-binding protein) and ceruloplasmin (copper-binding protein) are decreased in children with autism. Decreased blood levels of reduced glutathione, glutathione peroxidase (GSH-Px), methionine and cysteine, and increased oxidized glutathione were reported in autistic patients in comparison with healthy subjects [11]. Activity of erythrocyte superoxide dismutase (SOD), erythrocyte, and plasma GSH-Px in children with autism were significantly lower than in subjects with standard development. These results indicate that children with autism have low level of blood antioxidant enzyme systems [9, 12]. Increased antioxidant enzymes activities (SOD and xantine oxidase) may reflect a previous cellular oxidative stress or serve as a compensatory mechanism. The increased activity of erythrocyte SOD and decreased catalase activity may result in overproduction of hydrogen peroxide (H_2_O_2_) in erythrocytes [8].

Brain mitochondria play an important role in generation of free radicals and ATP production. A key component of the respiratory chain in mitochondria is Coenzyme Q_10_ (CoQ_10_), an essential factor for cell bioenergetics. As an antioxidant it decreases oxidative stress. Our previous study determined baseline levels of CoQ_10-OX _, CoQ_9-OX_ in various rat brain regions and proved the bioavailability of the liposomal ubiquinol to selected brain regions after its administration into right brain ventricle. Our data indicate that administration of ubiquinol may create the basis for modulation of neuronal activities in specific brain regions [13]. Based on these results, we expected beneficial effects of ubiquinol supportive therapy in children with autism—behavior, physiological functions, antioxidant status, and lipid peroxidation.

## 2. Materials and Methods

### 2.1. Children with Autism

Twenty four children (17 boys, 7 girls; with male to female ratio 2.4 : 1), aged 3–6 years, were included in the study according to the criteria of DSM IV (Diagnostic and Statistical Manual of Mental Diseases, USA) and using CARS (Childhood Autism Rating Scale, a test combining parent reports and direct observation by the professional) [14]. All parents signed an informed consent with including their children to this project. Children were coming from home settings. A placebo group of children with autism was not included in the study for ethical reason.

### 2.2. Psychological Test

Before the beginning of the study, all children with neurology disturbances were examined by psychologists, or neurologists. Children with obesity, diabetes, or epilepsy were not included in the study. Parents provided data about behavior of their children before and after three months of ubiquinol supplementation (Table 1). The screening tool was designed by a psychologist specifically for the purposes of the presented study.

### 2.3. Ubiquinol Treatment

Liquid liposomal ubiquinol (Li-QH—liposomal liquid reduced Coenzyme Q_10_, produced by Tishcon corp., Westbury, New York, USA) was diluted in milk, tea, or juice.

Children were supplemented during the first week at a daily dose of 50 mg ubiquinol. Evidence indicates beneficial effects of treatment with higher doses; therefore after the first week the daily dose was then increased to 2 × 50 mg ubiquinol (morning and at lunch with meal). If the children were hyperactive, time of application was changed: 50 mg ubiquinol morning and 50 mg ubiquinol early in the evening before sleeping instead of at lunch time. Discontinuation of treatment after two months was in children with asthma (1), broken leg (1), increased activity (1), aggression (2), and sleep disorder (1). Children with autism were treated with last dose of ubiquinol 24 hours before blood collection.

### 2.4. Biochemical Study

*Plasma samples* for estimation of TBARS, CoQ_10-TOTAL_, *α*-tocopherol, *γ*-tocopherol, and *β*-carotene were prepared from blood and collected into tubes with heparin after centrifugation at 1.96 ×g for 15 minutes.

### 2.5. Determination of TBARS in Plasma

Thiobarbituric acid reactive substances (TBARS) in plasma were determined spectrophotometrically by the method in [15].

### 2.6. Determination of *CoQ*_10-TOTAL_, *γ*-Tocopherol, *α*-Tocopherol, and *β*-Carotene Concentrations

Antioxidants (CoQ_10-TOTAL_, *γ*-tocopherol, *α*-tocopherol, and *β*-carotene) concentrations in the plasma were determined simultaneously by isocratic high-performance liquid chromatography (LKB, Sweden) according to [16, 17]. Oxidation of ubiquinol was performed before analysis [18]. Plasma samples (500 *μ*L) were extracted by mixture hexane/ethanol (5/2, v/v; Merck). Organic layer was evaporated under nitrogen, and the residue dissolved in 99.9% ethanol and injected on the 7 *μ*m column SGX C18 (Tessek, Czech Republic). Elution was performed with methanol/acetonitril/ethanol (6/2/2; v/v/v; Merck) and flow rate 0.85 mL/min. Concentrations of CoQ_10-OX_ were detected spectrophotometrically at 275 nm, *γ*-tocopherol and *α*-tocopherol at 295 nm, and *β*-carotene at 450 nm, using external standards (Sigma; Germany). Data were collected and processed using CSW32 chromatographic station (DataApex Ltd; Czech Republic).

Concentration of antioxidants (CoQ_10-*Total*_, *γ*-tocopherol, *α*-tocopherol, and *β*-carotene) and lipid peroxidation were determined in plasma before and after three months of supportive therapy with ubiquinol.

### 2.7. Statistics

Statistical analysis of the data was made using paired Student's *t*-test. Differences between baseline values and after three months of treatment were considered statistically significant if corrected *P* < 0.05.

## 3. Results

Mean plasma TBARS and antioxidants (*α*-tocopherol and *β*-carotene) concentrations in children with autism were in reference range both at the baseline and also after three months of ubiquinol treatment in comparison. Baseline *γ*-tocopherol concentrations were slightly below reference range and increased after treatment. Mean baseline concentration of CoQ_10-TOTAL_ was in reference values. After three months of ubiquinol treatment concentrations of CoQ_10-TOTAL_ were in the range of 1.633–5.496 *μ*mol/L. Significant improvement in autistic symptomology was observed after three months of ubiquinol supplementary therapy in children prevailingly at over 2.5 *μ*mol/LCoQ_10-TOTAL_ plasma concentration (Table 2). Based on parents reports during the supportive therapy improved communication with parents was observed in 12% of children, playing games with friends in 42%, verbal communication in 21%, and sleeping in 34%, and food rejection decreased in 17%, aggressiveness decreased in 13%, and self-harm decreased in 14% of the subjects (Table 1). In rare cases, especially in the first days of ubiquinol supplementation, opposite effects, such as increased destructive manifestations of anger, hyperactivity, and sleep disorders were observed. These cases were managed by modifying the time of ubiquinol treatment (50 mg ubiquinol morning and 50 mg ubiquinol early in the evening before sleeping).

## 4. Discussion

Children with autism are more vulnerable to *oxidative stress*; lipid peroxidation markers are elevated [9, 19]. Frustaci et al. reviewed data on TBARS (the end product of lipid peroxidation in the body) in patients with autism. Its plasma concentration in autism is controversial, from increasing over control values or decreasing in comparison with control values [11]. Our previous study showed differences in baseline values of TBARS, depending on age. In 44 children (34 boys and 10 girls), aged 3–10 years, plasma level of TBARS was in reference values (4.262 ± 0.085 *μ*mol/L), while in 12 children (9 boys and 3 girls), aged 11–18 years, TBARS levels were significantly higher (4.624 ± 0.177 *μ*mol/L, *P* < 0.05) in comparison with reference values. Ratio of male to female was 3 : 1. The presented study shows plasma TBARS concentration in reference values of children with autism aged 3–6 years (Table 2). Several factors could contribute to the TBARS plasma level in patients with autism, as age, metabolic changes, and mitochondrial disturbances of brain and skeletal muscle. In the study obese children with autism, children with diabetes, or epilepsy were not included. Baseline values of antioxidants and TBARS were compared with reference values. Control group and placebo group of children with autism were not included in the study for ethical reason. Additional studies with estimation of oxidative stress parameters in patients with autism are warranted.

*Antioxidant *defence systems and mitochondrial dysfunction are included in pathobiochemical mechanism of autism [4, 10]. Zoroglu et al. [8] documented increased antioxidant enzymatic activities; other authors published low level of blood antioxidant enzyme system in autistic children. Krajcovicova-Kudlackova et al. [20] published insufficient plasma concentrations of vitamins E and A and lycopene in children with autism. In our study baseline values of plasma antioxidants in children with autism as *α*-tocopherol,*β*-carotene, and CoQ_10-TOTAL_ were in reference values (Table 2).

If abnormalities of a dynamic balance between antioxidants and reactive oxygen radicals production are present in brain, free radicals accumulation could damage brain tissue [9, 12]. In children with autism (aged 4–10 years) significantly lower levels of mitochondrial respiratory chain proteins were found, as in complex III and V in cerebellum, complex I in frontal cortex, and complexes II, III, and V in the temporal cortex. Chauhan et al. [21] observed that deficits in respiratory chain complexes in children with autism may readjust to normal levels by adulthood. Mild mitochondrial dysfunction manifested as carnitine deficiency accompanied by elevation in lactate, alanine, and ammonia levels was observed in autism [22]. Mitochondrial dysfunction with increased membrane degradation, impairment of electron transport chain activity, and decreased synthesis of ATP was observed in the brain [6] and in muscle biopsy in children with autism [23]. Classical mitochondrial diseases occur in individuals with autism and are usually caused by genetic anomalies of mitochondrial respiratory pathways [24]. In lymphocytic mitochondria decreased activity of complex I, higher mitochondrial rate of hydrogen peroxide production mtDNA overreplication, and mtDNA deletions were found in children with autism [3].

Elevated lactate, pyruvate, lactate/pyruvate ratio, alanine, creatine kinase, ammonia, aspartate aminotransferase, alanine aminotransferase activities, and low total carnitine belong to the abnormal biomarkers of mitochondrial dysfunction in autism [1]. Early assessment of antioxidants status would have better prognosis as it may decrease the oxidative stress before inducing irreversible brain damage [19].

Nutrition supplementation is used in autism, such as vitamin C, carnosine, zinc, reduced glutathione, fish oil (rich in EPA), melatonin, and vitamin B6 in combination with Mg [6]. Treatment also includes acetylcholin esterase inhibitors, music and movement therapy [25], carnitine, hyperbaric oxygen treatment [2], immunomodulation and anti-inflammatory treatment, oxytocin, and vision therapy, multivitamin/mineral complex, PUFA, elimination diets, acupuncture, auditory integration training, and massage [26]. In review [1] authors proposed possible antioxidant treatment with carnitine, CoQ_10_, and high doses of B-vitamins. In our study, the best improvement in behavioral and psychological functions was observed after three months of ubiquinol supportive therapy in children with autism at over 2.5 *μ*mol/LCoQ_10-TOTAL_ plasma concentration, which is used as curative value [27].

**Figure 1:**
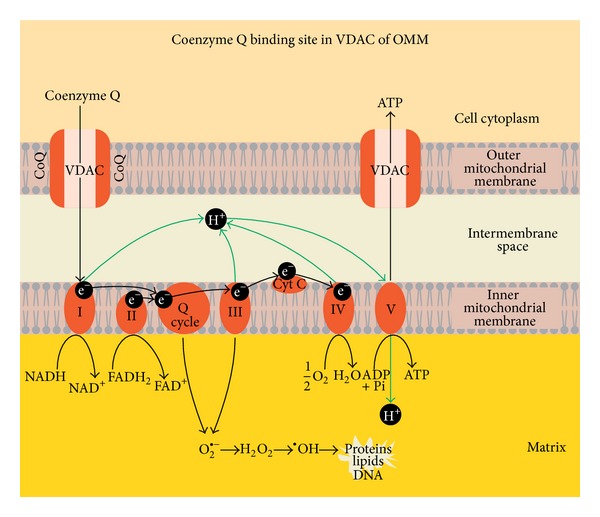
Proposed novel Coenzyme Q binding site in Voltage Dependent Anion Channel of outer mitochondrial membrane. CoQ—Coenzyme Q; VDAC—Voltage Dependent Anion Channel; ATP—adenosine triphosphate; ADP—adenosine diphosphate; Pi—inorganic phosphate; I, II, III, IV, and V—respiratory chain complexes; H^+^—proton; e^−^—electron; Q-cycle—Coenzyme Q cycle; cyt c—cytochrome c; NADH—reduced nicotinadenindinucleotid; NAD^+^—nicotinadenindinucleotid; FADH_2_—reduced flavinadenindinucleotid; FAD—flavinadenindinucleotid; O_2_^∙−^—superoxide radical; H_2_O_2_—hydrogen peroxide; OH^∙^—hydroxyl radical; H_2_O—water; O_2_—oxygen.

Mitochondrial Coenzyme Q functions include regulation of electron transport in the respiratory chain, receiving electrons from complex I, complex II, and passing them to complex III, and transfer of protons from fatty acids to matrix. As an alternative Coenzyme Q function is possible in regulation of permeability transition pore opening and nutrition uptake through the Voltage Dependent Anion Channel (VDAC) of outer mitochondrial membrane (Figure 1). We assume that ubiquinol supportive therapy may improve brain mitochondrial function and ATP production and affect brain oxidative stress. The mechanism by which ubiquinol decreases symptoms in children with autism is not fully known.

## 5. Summary

Beneficial effect of ubiquinol in children with autism has been demonstrated for the first time. We assume that plasma concentration of CoQ_10-TOTAL_ and lipid peroxidation could be used as important biomarkers of ubiquinol supportive therapy. Additional study with a larger number of patients is warranted to confirm these promising findings.

## Figures and Tables

**Table 1:** Effect of ubiquinol on psychological functions and behavior in children with autism.

	Improving (%)	Deterioration (%)
Hyperactivity	7	4
Aggression	13	7
Self-harm	14	0
Toys destruction	19	3
Playing with friend	42	4
Communication with parents	12	0
Verbal communication	21	0
Food rejection	17	0
Sleep disorders	34	8

**Table 2:** Effect of ubiquinol on TBARS and antioxidants in plasma of children with autism (in µmol/L).

	Reference values	Baseline values	Ubiquinol (3 months)
TBARS	<4.50	4.380 ± 0.153	4.200 ± 0.241
*γ*-Tocopherol	2.00–7.00	1.625 ± 0.160	1.927 ± 0.304
*α*-Tocopherol	15–40	17.98 ± 0.920	19.14 ± 0.872
*β*-Carotene	0.30–3.00	0.983 ± 0.164	0.804 ± 0.129
CoQ_10-TOTAL_	0.40–1.00	0.512 ± 0.164	3.016 ± 0.307*

µmol/L: micromol/liter; TBARS: thiobarbituric acids reactive substances; *γ*-tocopherol: gamma-tocopherol; *α*-tocopherol: alpha-tocopherol; CoQ_10-TOTAL_ (ubiquinol + ubiquinone); data are expressed as mean ± standard error of the mean; *P < 0.0001.
