# Expression of Concern on “Ubiquinol Improves Symptoms in Children with Autism”

**DOI:** 10.1155/2020/3803527

**Published:** 2020-12-19

**Authors:** Oxidative Medicine and Cellular Longevity

*Oxidative Medicine and Cellular Longevity* would like to express concern with the article titled “Ubiquinol Improves Symptoms in Children with Autism” [1] due to issues with the design and reporting of this clinical study.

Ethical approval was not obtained although this appears to be a prospective clinical test of ubiquinol, i.e. a clinical trial, and the study was not registered in a clinical trials registry. The small sample size of 24 patients indicates that this is a phase 1 clinical trial, which can only inform the safety and not the efficacy of the compound in autism. The research was funded by Tishcon Corp, who market products that contain ubiquinol, and this was not stated as a conflict of interests though it is disclosed in the funding section.

We have asked the authors' institution to formally investigate.
